# Pharmacokinetic rationale for the interaction of 5-fluorouracil and misonidazole in humans.

**DOI:** 10.1038/bjc.1983.253

**Published:** 1983-11

**Authors:** B. J. McDermott, H. W. Van den Berg, W. M. Martin, R. F. Murphy

## Abstract

As part of a Phase I clinical trial, 5 patients received 5-fluorouracil (FU) both singly and in combination with misonidazole (MISO) for the treatment of gastrointestinal cancer. Concentrations of total FU and F-containing metabolites in urine specimens, taken during 48 h after therapy, were determined. The clearance of FU following administration of 1.0 or 1.5 g FU m2 was significantly reduced by treatment with MISO (1.75-2.0 gm-2) given 2 h prior to FU therapy. Reduced clearance of FU by MISO was associated with an earlier onset of the period of nonlinearity of FU pharmacokinetics and an increased half-life of elimination. Furthermore, the clearance of FU correlated inversely with the severity of gastrointestinal toxicity. The mechanism of MISO enhancement of FU action is unlikely to be competition for microsomal enzymes, as proposed for the interaction of MISO and alkylating agents, since FU is catabolized at mitochondrial and cytosolic sites.


*Br. J. Cancer* (1983), **48**, 705–710

### Pharmacokinetic rationale for the interaction of 5-fluorouracil and misonidazole in humans

B.J. McDermott[1]*, H.W. Van den Berg[2], W.M.C. Martin[3] & R.F. Murphy[1]

[1]*Department of Biochemistry,* [2]*Therapeutics and Pharmacology, The Queen's University of Belfast, BT9 7BL and* [3]*Northern Ireland Radiotherapy Centre, Belvoir Park Hospital, Belfast BT8 8JR.*

**Summary** As part of a Phase I clinical trial, 5 patients received 5-fluorouracil (FU) both singly and in combination with misonidazole (MISO) for the treatment of gastrointestinal cancer. Concentrations of total FU and F-containing metabolites in urine specimens, taken during 48 h after therapy, were determined. The clearance of FU following administration of 1.0 or 1.5 g FU $m^{-2}$ was significantly reduced by treatment with MISO (1.75–2.0 g $m^{-2}$) given 2 h prior to FU therapy. Reduced clearance of FU by MISO was associated with an earlier onset of the period of nonlinearity of FU pharmacokinetics and an increased half-life of elimination. Furthermore, the clearance of FU correlated inversely with the severity of gastrointestinal toxicity. The mechanism of MISO enhancement of FU action is unlikely to be competition for microsomal enzymes, as proposed for the interaction of MISO and alkylating agents, since FU is catabolized at mitochondrial and cytosolic sites.

There is considerable evidence from *in vitro* and *in vivo* studies in animals to suggest that the 2-nitroimidazole, misonidazole (MISO), a radiosensitizer of hypoxic cells, enhances tumour response to chemotherapy (reviews by McNally, 1982; Millar, 1982; Siemann, 1982*a*). The potentiation mechanism, however, is unclear (reviewed by Brown, 1982) and clinical studies (Spooner *et al.*, 1982; Urtasun *et al.*, 1982) are limited.

Administration of MISO simultaneously with 5-fluorouracil (FU) in mice bearing Lewis lung carcinoma enhanced the tumour response assessed by clonogenic cell survival (Stephens *et al.*, 1981). FU-induced growth delay of the 16/C mammary carcinoma and the KHT sarcoma was increased by the addition of MISO (Tannock, 1980*a*). Host toxicity, measured by death and loss of body weight, however, was also increased, resulting in only a small gain in therapeutic index.

Since the enhancement of effect by MISO occurs with cytotoxic drugs of diverse mechanisms of action, Stephens *et al.* (1981) suggested that MISO may alter drug pharmacokinetics, leading to greater exposure to the drug. In animal studies, MISO has been shown to alter the pharmacokinetics of alkylating agents (Tannock, 1980*b*; Stephens *et al.*, 1981; Clutterbuck *et al.*, 1982; Workman *et al.*, 1983) and nitrosoureas (Tannock, 1980*b*; Lee & Workman, 1983), but the effect of the sensitizer on drug elimination in humans has not been reported.

*Present address: Clinical Oncology Unit, University of Bradford, Bradford BD7 1DP.
Correspondence: B.J. McDermott
Received 18 March 1983; accepted 22 July 1983.

This paper describes a preliminary study of the pharmacokinetics of FU and of FU with concurrent administration of MISO for gastrointestinal cancer as part of a Phase I clinical trial.

### Materials and methods

### *Patients and protocols*

Five patients with advanced gastrointestinal cancer (Table I) participated with informed consent. Subjects were evaluated by clinical examination, biochemical tests for liver, renal and haematological function, chest and skeletal radiography and radioisotope scans. Patients received: FU (1.0 g $m^{-2}$); MISO (1.75 or 2.0 g $m^{-2}$) and FU (1.0 g $m^{-2}$); FU (1.5 g $m^{-2}$); MISO (1.75 or

**Table I** Patient protocols

| *Patient* | *Sex* | *Age* | *Surface area ($m^2$)* | *Disease status* |
|---|---|---|---|---|
| R.C. | M | 42 | 1.7 | Carcinoma of rectum, resected; lung metastases |
| J.K. | M | 61 | 1.7 | Carcinoma of colon, liver metastases |
| M.P. | F | 72 | 1.3 | Carcinoma of rectosigmoid, peri-anal recurrence |
| S.A. | F | 70 | 1.5 | Carcinoma of sigmoid colon, local recurrence |
| R.J. | M | 68 | 1.8 | Carcinoma of caecum, resected; small liver metastases |

© The Macmillan Press Ltd., 1983

2.0 g m$^{-2}$) and FU (1.5 g m$^{-2}$); consecutively. Each course of MISO and FU was administered 3–5 weeks after the corresponding treatment with FU alone. MISO was administered orally 2 h before FU, which was given by i.v. injection. Patients were questioned regarding possible side-effects, in particular, nausea and vomiting, alopecia and neuropathy. Sodium azide (1 g l$^{-1}$) was used as preservative for urine specimens which were collected as voided spontaneously, before therapy and at timed intervals up to 48 h after treatment. The volumes of specimens were recorded and aliquots (25 ml) were stored at −20°C.

*Analysis*

Total FU and F-containing metabolites were estimated in urine using a F-specific electrode after combustion of specimens in an oxygen flask (McDermott *et al.*, 1982). Analytical recovery of the method was 99.5% and 96.3%. and intra-assay variation (s.e.) was 2.5% ($n=8$) and 2.1% ($n=12$) for FU concentrations of $2.0\times10^{-5}$ M and $9.0\times10^{-4}$ M, respectively. Inter-assay variation (s.e.) for analysis of $9.0\times10^{-4}$ M FU was 3.3% ($n=8$).

The time courses of total levels of drug (i.e. drug plus metabolites) were evaluated by the "Sigma-minus" method (Wagner, 1963), which consists of plotting the logarithm of ($Xu^{\infty}-Xu^{t}$) against time, $t$, where ($Xu^{\infty}-Xu^{t}$) represents the sum of the amounts of total drug excreted until excretion may be considered to be complete minus the cumulative amount of drug excreted to $t$. The data are presented as % dose excreted remaining in the body to $t$, i.e. log ($Xu^{\infty}-Xu^{t}$) $\times 100/Xu^{\infty}$. Profiles of total drug concentrations can be used to obtain pharmacokinetic parameters of it is assumed that the rate constants of elimination for all primary metabolites are appreciably greater than the rate constant ($\beta$) for elimination of the parent drug (Gibaldi & Perrier, 1975). When drug elimination occurs by first-order processes, the "Sigma-minus" plot is linear with slope, $-\beta/2.303$. Half-lives of FU elimination ($t_{\frac{1}{2}}$) were estimated from the initial apparently linear portions of the plots and were calculated as $0.693/\beta$. The areas under "Sigma-minus" curves (AUC) were determined using the trapezoid rule (Gibaldi & Perrier, 1975).

Statistical variations were expressed as standard errors of the mean (s.e.) and comparisons were made using the Student's $t$-test for paired samples.

### Results

The elimination of total drug for each patient after the administration of different dosages of FU alone and of FU with MISO is shown in Figure 1 a–e. The pharmacokinetic profiles are composed of linear and nonlinear kinetics as demonstrated previously for the elimination of FU in patients receiving therapeutic doses for breast cancer (McDermott *et al.*, 1982). After administration of 1.0 g FU m$^{-2}$, enhancement of the extent of saturation of drug elimination by MISO was particularly noticeable in 3 patients (Figure 1 a, d, e) but an opposite effect was also observed (Figure 1c). MISO amplified the nonlinearity of FU elimination kinetics in 2 patients given 1.5 g FU m$^{-2}$ (Figure 1 c, e).

Figure 2 shows the kinetic characteristics of the elimination profiles in terms of AUC values and the percentages of total dose excreted in 48 h. MISO had a potentiating effect on the amount of drug remaining in the body in 9/10 courses of treatment investigated (Figure 2). Prior administration of MISO with FU doses of 1.0 or 1.5 g m$^{-2}$ led to mean increases in AUC of 91.5±31.1 (s.e.) %h$^{-1}$ and 103.4±37.5 (s.e.) % × h, respectively, which were significant ($P<0.05$). The delayed clearance of FU from the body in the presence of MISO was reflected in a reduced extent of urinary excretion of total drug by only 1 patient after dosage of 1.0 g FU m$^{-2}$ but by all patients given 1.5 g FU m$^{-2}$. The average reduction in the excretion of the higher dosage of FU was 20.4±6.8 (s.e.) %, which was significant ($P<0.05$). $t_{\frac{1}{2}}$ value of drug elimination increased when MISO was administered with FU at both levels of dosage and the average $t_{\frac{1}{2}}$ values was greater after FU dosage of 1.5 g m$^{-2}$ than of 1.0 g m$^{-2}$ (Table II), but these results were not statistically significant.

No toxic effects were evident after therapy with FU doses of 1.0 g m$^{-2}$. Gastrointestinal symptoms were the most common sign of toxicity and these occurred during 8 of the treatments with the higher dosage of FU or with the combined MISO and FU therapy. MISO potentiated the severity of side-effects during 5 courses of therapy. Table III shows the relationship between the degree of gastro-intestinal disturbance and FU clearance. Patient R.J. had the highest AUC value, after

**Table II** $t_{\frac{1}{2}}$ values for the elimination of different dosages of FU, with and without MISO

| | $t_{\frac{1}{2}}(h)$ (*mean** ± *s.e.*) |
|---|---|
| FU (1.0 g m$^{-2}$) | 2.84±0.38 |
| FU (1.0 g m$^{-2}$)+MISO | 3.30±0.40 |
| FU (1.5 g m$^{-2}$) | 3.70±0.21 |
| FU (1.5 g m$^{-2}$)+MISO | 4.32±0.54 |

*Average value of 5 patients.

**Figure 1** "Sigma-minus" plots of total drug excreted after administration of 1.0 mg FU $m^{-2}$ (circles) and 1.5 mg FU $m^{-2}$ (squares) alone (open symbols) or in conjunction with MISO (closed symbols) to patients (a) R.C. (b) J.K. (c) M.P. (d) S.A. and (e) R.J.

administration of FU (1.5 g $m^{-2}$) with MISO (2.0 g $m^{-2}$). In addition to experiencing the most severe gastrointestinal upset, this patient suffered alopecia and slight neuropathy.

**Table III** Incidence of gastrointestinal toxicity

| *No. of treatments* | *Gastrointestinal symptoms* | *Mean AUC value [range]* (%$h^{-1}$) |
|---|---|---|
| 12 | None | 495 (375–648] |
| 3 | Anorexia, mild nausea | 560 [471–682] |
| 4 | Severe nausea, some vomiting | 712 [625–796] |
| 1 | Severe vomiting | 892 |

### Discussion

The hypothesis that the enhancement by nitroimidazole radiosensitizers of tumour response to chemotherapeutic agents is mainly a result of alterations in drug pharmacokinetics has been proposed by some authors (Tannock, 1980*b*; Stephens *et al.*, 1981; Clutterbuck *et al.*, 1982; Workman & Twentyman, 1982; Lee & Workman, 1983; Workman *et al.*, 1983) but questioned by others (Martin *et al.*, 1981; Hirst *et al.*, 1982; Mulcahy *et al.*, 1982; Murray & Meyn, 1983). The doses of MISO used in experimental studies generally produce peak plasma levels of MISO 5 to 10 times greater than can be achieved in humans (reviewed by Workman, 1980). The plasma $t_{\frac{1}{2}}$ of

**Figure 2** Clearance parameters of total drug after administration of FU alone (□) and in combination with MISO (▧). The values included in the upper diagram represent the % increases in AUC produced by the addition of MISO to FU treatment. Normal ranges of creatinine clearance (C.C.): males, 85–120 ml min$^{-1}$; females, 75–115 ml min$^{-1}$.

MISO in man, however, is 10–20 times greater than in the mouse (Workman, 1980). By administering multiple small doses of MISO to mice to produce the prolonged low concentrations that can be achieved safely in man, enhancement of the tumour cytotoxicity of alkylating agents has been demonstrated (Brown & Hirst, 1982; Twentyman & Workman, 1983).

The findings of the present study are an initial indication that MISO may influence drug response in man and that the interaction has a pharmacokinetic basis. MISO produced enhancement of AUC values from 2–33% of those observed when FU was administered alone, during all but one treatment (Figure 2). The sensitizer amplified the extent of saturations of FU elimination during 5 courses of treatment (Figure 1). The failure to observe changes in the shape of some of the "Sigma-minus" curves with MISO therapy could be explained by the fact that increases in AUC of these semilogarithmic plots are less apparent as the extent of saturated elimination increases. As a result of increased saturation at the higher dosage of FU (1.5 g m$^{-2}$), there was a significant reduction in excretion of total drug by MISO (Figure 2), but this was less apparent at the lower dosage (1.0 g m$^{-2}$). Also, there was a tendency for the apparent $t_{\frac{1}{2}}$ values to increase as a consequence of earlier onset of nonlinear pharmacokinetics with increased dosage of FU and/or the addition of MISO (Table II), although these changes were not statistically significant.

The anomalous values of excretion parameters (Figure 2) may be consequences of changes in clinical condition. The increased excretion by Patient R.C. at the lower dosage of FU with MISO correlated with an abnormally high value of creatinine clearance on the day of therapy. The limited extent of urinary excretion of total drug by Patient J.K. may be a result of secondary disease in the liver. In addition, increasingly efficient catabolism associated with reduction of the volume of liver metastases could account for the decreased AUC with escalation of FU dose given alone and the reduction in AUC in the presence of MISO at the higher FU dosage.

Clearance of FU correlated inversely with the degree of gastrointestinal side-effects (Table III), which were potentiated by MISO in 50% of cases. However, the toxicity to normal tissues was mild, compared with other standard regimes of cytotoxic therapy. Only Patient R.C. with pulmonary metastases had a measurable lesion, but the metastases did not respond to FU, at either dose or with MISO. Two patients, S.A. and M.P., received local radiotherapy concomitantly with cytotoxic drugs and the remaining 2 patients, J.K. and R.J., had intra-abdominal tumours, which were not measurable clinically.

The mechanisms by which MISO may alter the disposition of chemotherapeutic agents is not well understood. The potentiating effect of MISO may be a consequence of competition for catabolic sites with other drugs which are metabolized by microsomal oxidation (Workman & Twentyman, 1982; Siemann, 1983; Workman *et al.*, 1983) but there are conflicting results (Law *et al.*, 1981; Siemann, 1982*b*). The present study shows that modulation of the pharmacokinetics of cytotoxic drugs by MISO cannot be simply competition for microsomal enzymes, since FU is catabolized at mitochondrial and cytosolic locations (Wasternak, 1979). Clearance values of FU and its primary catabolite, 5′6′-dihydro-5-fluorouracil (DHFU), derived from their concentrations in plasma showed that saturated elimination could be observed within 6 h of drug administration (McDermott *et al.*, 1982). These data suggested that the initial catabolic step of FU degradation was saturable. Preliminary results of the influence of MISO on plasma levels of FU and DHFU have demonstrated a decreased ratio of FU to DHFU clearance after MISO administration suggesting that MISO itself may inhibit the initial catabolic step of FU metabolism. However, the present study has demonstrated a later onset of saturated kinetics of renal clearance of total drug which is influenced by MISO (Figure 1), indicating that an additional mechanism of inhibition of FU clearance may be implicated. Further studies are required to evaluate the relative importance of MISO in inhibiting either hepatic metabolism of FU or renal elimination of parent drug and metabolites.

### References

BROWN, J.M. (1982). The mechanisms of cytotoxicity and chemosensitization by misonidazole and other nitroimidazoles. *Int. J. Radiat. Oncol. Biol. Phys.*, **8**, 675.

BROWN, J.M. & HIRST, G.D. (1982). Effect of clinical levels of misonidaxole on the response of tumour and normal tissues in the mouse to alkylating agents. *Br. J. Cancer*, **45**, 700.

CLUTTERBUCK, R.D., MILLAR, J.L. & McELWAIN, T.J. (1982). Misonidazole enhancement of the action of BCNU and melphalan against human melanoma xenografts. *Am J. Clin. Oncol.*, **5**, 73.

GIBALDI, M. & PERRIER, D. (1975). *Pharmacokinetics*. New York, Marcel Dekker, Inc.

HIRST, D.G., BROWN, J.M. & HAZLEHURST, J.L. (1982). Enhancement of CCNU cytotoxicity by misonidazole: possible therapeutic gain. *Br. J. Cancer*, **46**, 109.

LAW, M.P., HIRST, D.G. & BROWN, J.M. (1981). Enhancing effect of misonidazole on the response of the RIF-1 tumour to cyclophosphamide. *Br. J. Cancer*, **44**, 208.

LEE, F.Y.F. & WORKMAN, P. (1983). Modification of CCNU pharmacokinetics by misonidazole—a major mechanism of chemosensitization in mice. *Br. J. Cancer*, **47**, 659.

McDERMOTT, B.J., VAN DEN BERG, H.W. & MURPHY, R.F. (1982). Nonlinear pharmacokinetics for the elimination of 5-fluorouracil after intravenous administration in cancer patients. *Cancer Chemother. Pharmacol.*, **9**, 173.

McNALLY, N.J. (1982). Enhancement of chemotherapy agents. *Int. J. Radiat. Oncol. Biol. Phys.*, **8**, 593.

MARTIN, W.M.C., McNALLY, N.J. & DE RONDE, J. (1981). Enhancement of the effect of cytotoxic drugs by radiosensitizers. *Br. J. Cancer*, **43**, 756.

MILLAR, B.C. (1982). Hypoxic cell radiosensitizers as potential adjuvants to conventional chemotherapy for the treatment of cancer. *Biochem. Pharmacol.*, **31**, 2439.

MULCAHY, R.T., SIEMANN, D.W. & SUTHERLAND, R.M. (1982). Nitrosourea misonidazole combination chemotherapy: effect on KHT sarcomas, marrow stem cell and gut. *Br. J. Cancer*, **45**, 835.

MURRAY, D. & MEYN, R.E. (1983). Enhancement of the DNA cross-linking activity of melphalan by misonidazole *in vivo*. *Br. J. Cancer*, **47**, 195.

SIEMANN, D.W. (1982*a*). Potentiation of chemotherapy by hypoxic cell radiation sensitizers—A review. *Int. J. Radiat. Oncol. Biol. Phys.*, **8**, 1029.

SIEMANN, D.W. (1982*b*). Response of murine tumours to combinations of CCNU with misonidazole and other radiation sensitizers. *Br. J. Cancer.* **45,** 272.

SIEMANN, D.W. (1983). Effect of pretreatment with phenobarbital or SKF 525A on the toxicity and antitumour activity of lomustine. *Cancer Treat. Rep.,* **67,** 259.

SPOONER, D., BUGDEN, R.D., PECKHAM, M.J. & WIST, E.A. (1982). The combination of 5-fluorouracil with misonidazole in patients with advanced colorectal cancer. *Int. J. Radiat. Oncol. Biol. Phys.,* **8,** 381.

STEPHENS, T.C., COURTENAY, V.D., MILLS, J., PEACOCK, J.H., ROSE, C.M. & SPOONER, D. (1981). Enhanced cell killing in Lewis lung carcinoma and a human pancreatic carcinoma xenograft by the combination of cytotoxic drugs and misonidazole. *Br. J. Cancer,* **43,** 451.

TANNOCK, I.F. (1980*a*). *In vivo* interaction of anti-cancer drugs with misonidazole or metronidazole: methotrexate, 5-fluorouracil and adriamycin. *Br. J. Cancer,* **42,** 861.

TANNOCK, I.F. (1980*b*). *In vivo* interaction of anti-cancer drugs with misonidazole or metronidazole: Cyclophosphamide and BCNU. *Br. J. Cancer,* **42,** 871.

TWENTYMAN, P.R. & WORKMAN, P. (1983). An investigation of the possibility of chemosensitization by clinically achievable concentrations of misonidazole. *Br. J. Cancer,* **47,** 187.

URTASUN, R.C., TANASICHUK, H., FULTON, D. & 5 others. (1982). Pharmacokinetic interaction of BCNU and misonidazole in humans. *Int. J. Radiat. Biol. Phys.,* **8,** 381.

WAGNER, J.G. (1963). Some possible errors in the plotting and interpretation of semilogarithmic plots of blood level and urinary excretion data. *J. Pharm. Sci.,* **52,** 1097.

WASTERNAK, C. (1979). Degradation of pyrimidines and pyrimidine analogs—pathways and mutual influences. *Pharmacol. Ther.,* **8,** 629.

WORKMAN, P. (1980). Pharmacokinetics of hypoxic cell radiosensitizers. A review. In: *Radiation Sensitizers,* (Ed. Brady), New York: Masson, p. 192.

WORKMAN, P. & TWENTYMAN, P.R. (1982). Enhancement by electron-affinic agents of the therapeutic effects of cytotoxic agents against the KHT tumour: structure-activity relationships. *Int. J. Radiat. Oncol. Biol. Phys.,* **8,** 623.

WORKMAN, P., TWENTYMAN, P.R., LEE, F.Y.F. & WALTON, M.I. (1983). Drug metabolism and chemosensitization. Nitroimidazoles as inhibitors of drug metabolism. *Biochem. Pharmacol.,* **32,** 857.


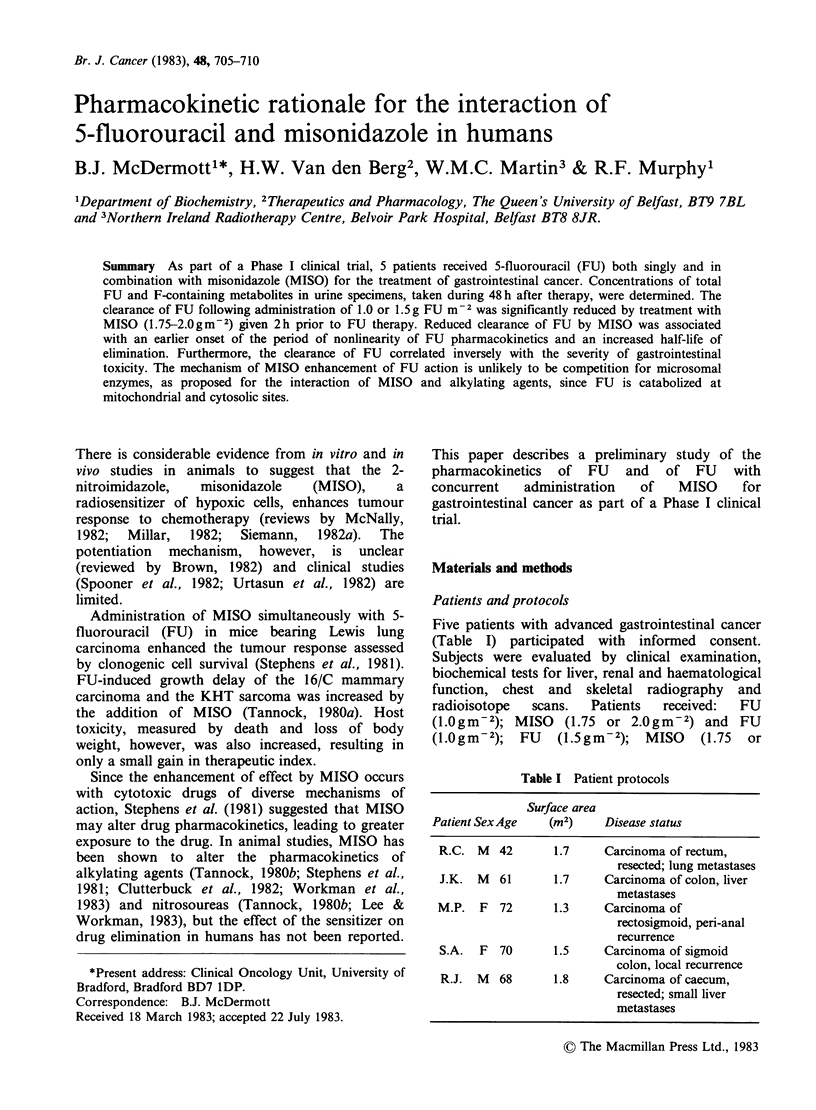

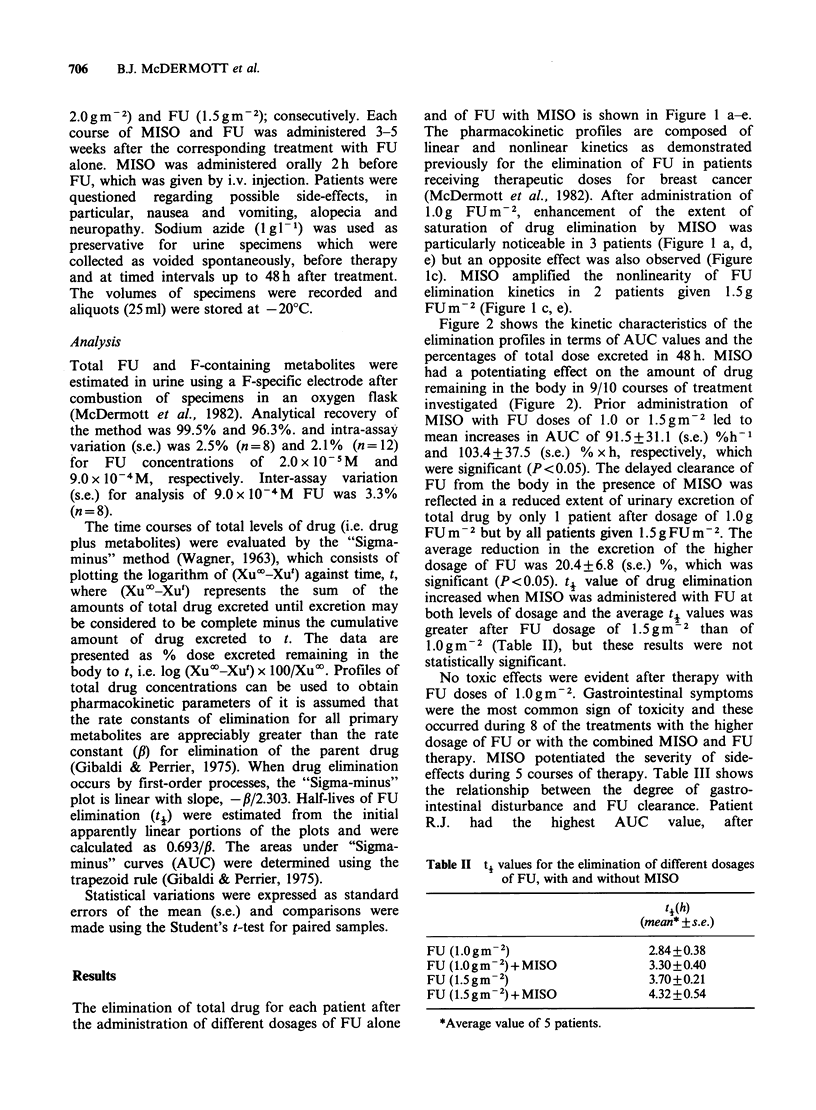

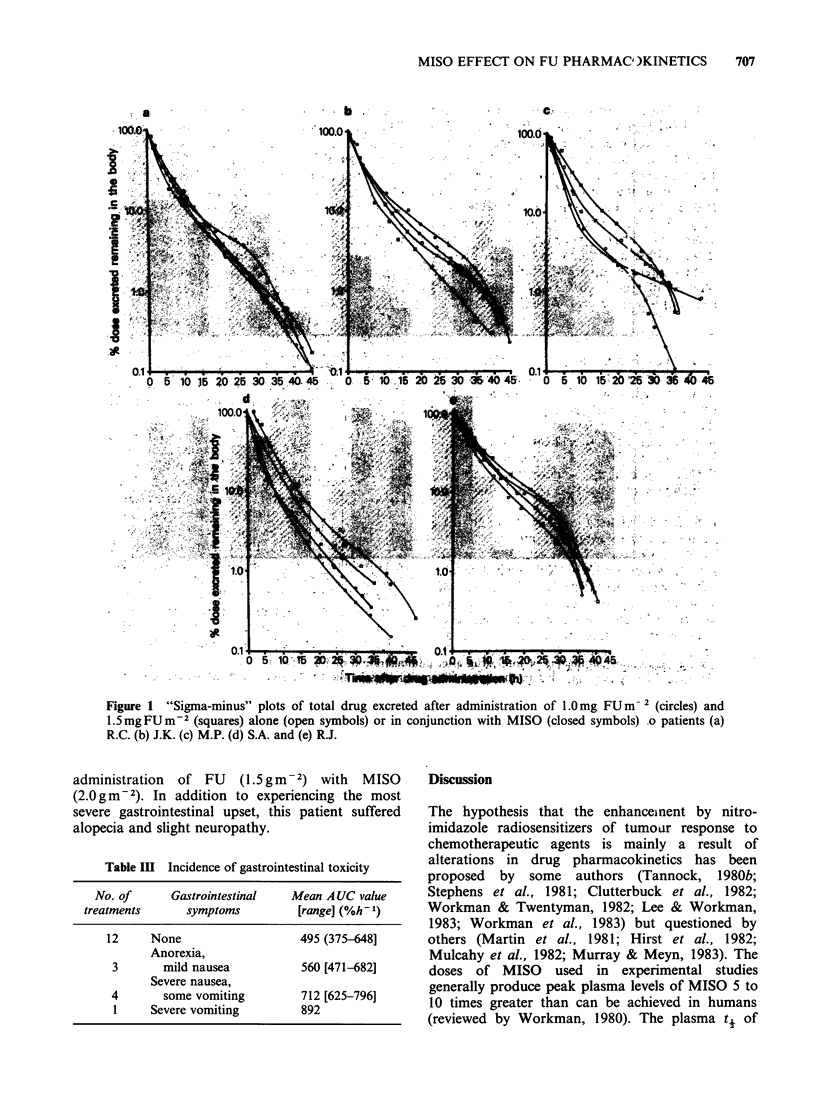

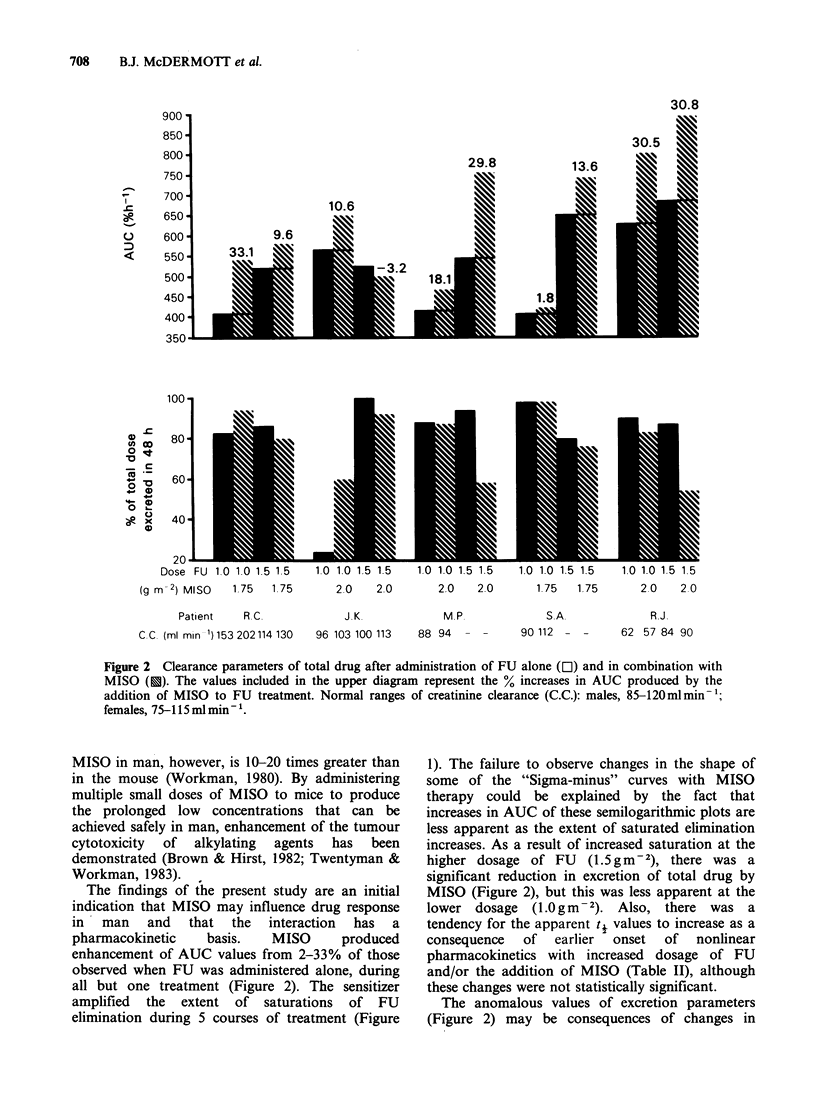

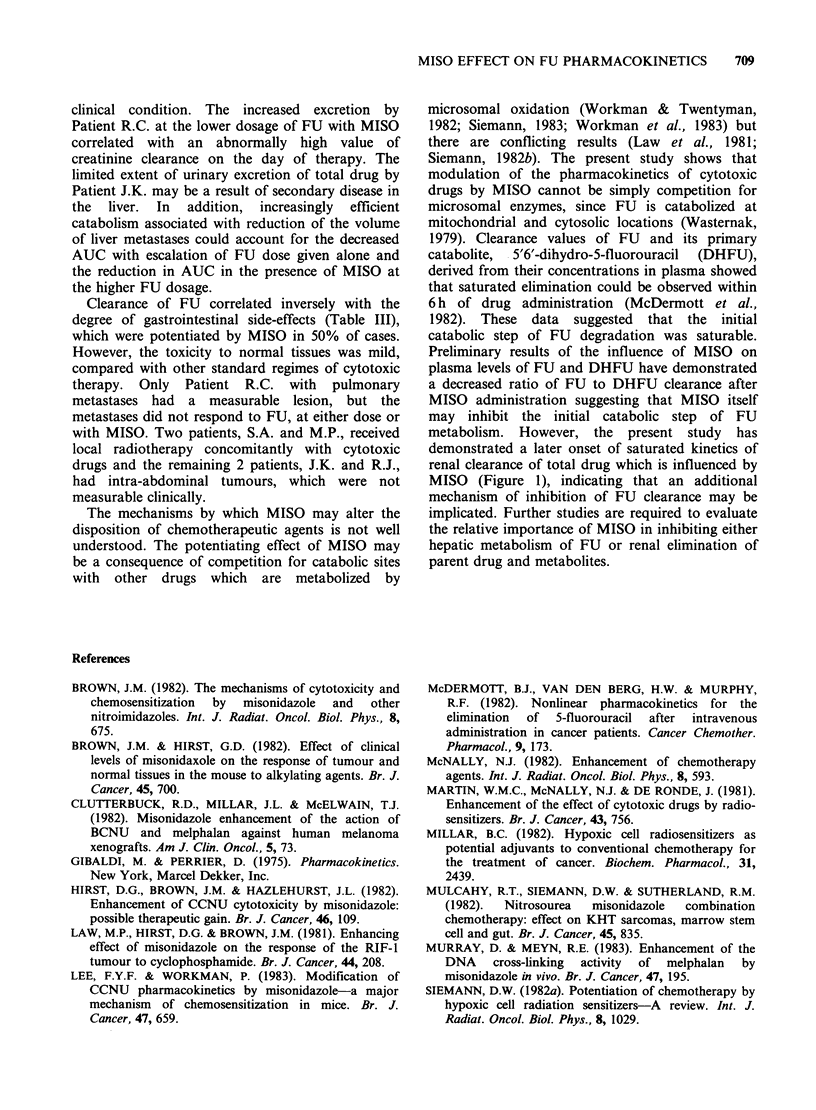

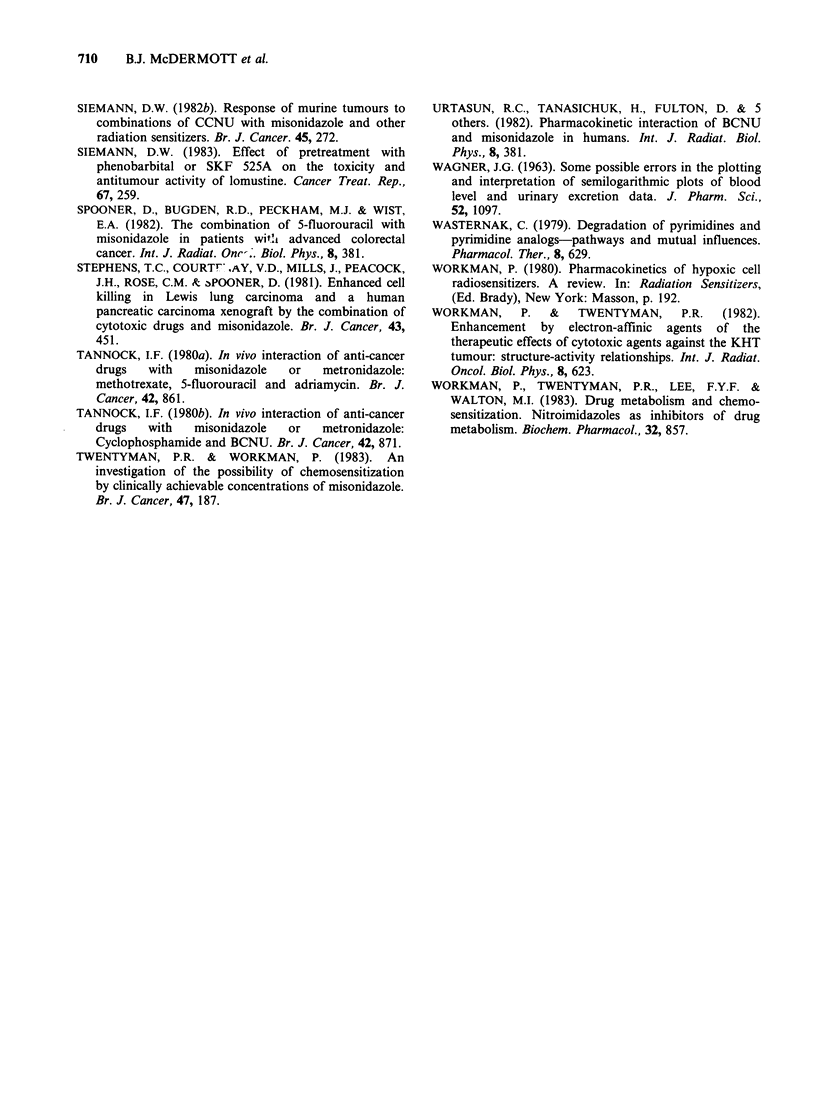


## References

[OCR_00508] Brown J. M., Hirst D. G. (1982). Effect of clinical levels of misonidazole on the response of tumour and normal tissues in the mouse to alkylating agents.. Br J Cancer.

[OCR_00502] Brown J. M. (1982). The mechanisms of cytotoxicity and chemosensitization by misonidazole and other nitroimidazoles.. Int J Radiat Oncol Biol Phys.

[OCR_00514] Clutterbuck R. D., Millar J. L., McElwain T. J. (1982). Misonidazole enhancement of the action of BCNU and melphalan against human melanoma xenografts.. Am J Clin Oncol.

[OCR_00524] Hirst D. G., Brown J. M., Hazlehurst J. L. (1982). Enhancement of CCNU cytotoxicity by misonidazole: possible therapeutic gain.. Br J Cancer.

[OCR_00529] Law M. P., Hirst D. G., Brown J. M. (1981). Enhancing effect of misonidazole on the response of the RIF-1 tumour to cyclophosphamide.. Br J Cancer.

[OCR_00534] Lee F. Y., Workman P. (1983). Modification of CCNU pharmacokinetics by misonidazole--a major mechanism of chemosensitization in mice.. Br J Cancer.

[OCR_00551] Martin W. M., McNally N. J., De Ronde J. (1981). Enhancement of the effect of cytotoxic drugs by radiosensitizers.. Br J Cancer.

[OCR_00540] McDermott B. J., van den Berg H. W., Murphy R. F. (1982). Nonlinear pharmacokinetics for the elimination of 5-fluorouracil after intravenous administration in cancer patients.. Cancer Chemother Pharmacol.

[OCR_00547] McNally N. J. (1982). Enhancement of chemotherapy agents.. Int J Radiat Oncol Biol Phys.

[OCR_00556] Millar B. C. (1982). Hypoxic cell radiosensitizers as potential adjuvants to conventional chemotherapy for the treatment of cancer.. Biochem Pharmacol.

[OCR_00562] Mulcahy R. T., Siemann D. W., Sutherland R. M. (1982). Nitrosourea-misonidazole combination chemotherapy: effect on KHT sarcomas, marrow stem cells and gut.. Br J Cancer.

[OCR_00568] Murray D., Meyn R. E. (1983). Enhancement of the DNA cross-linking activity of melphalan by misonidazole in vivo.. Br J Cancer.

[OCR_00585] Siemann D. W. (1983). Effect of pretreatment with phenobarbital or SKF 525A on the toxicity and antitumor activity of lomustine.. Cancer Treat Rep.

[OCR_00573] Siemann D. W. (1982). Potentiation of chemotherapy by hypoxic cell radiation sensitizers--a review.. Int J Radiat Oncol Biol Phys.

[OCR_00580] Siemann D. W. (1982). Response of murine tumours to combinations of CCNU with misonidazole and other radiation sensitizers.. Br J Cancer.

[OCR_00597] Stephens T. C., Courtenay V. D., Mills J., Peacock J. H., Rose C. M., Spooner D. (1981). Enhanced cell killing in lewis lung carcinoma and a human pancreatic-carcinoma xenograft by the combination of cytotoxic drugs and misonidazole.. Br J Cancer.

[OCR_00611] Tannock I. F. (1980). In vivo interaction of anti-cancer drugs with misonidazole or metronidazole: cyclophosphamide and BCNU.. Br J Cancer.

[OCR_00605] Tannock I. F. (1980). In vivo interaction of anti-cancer drugs with misonidazole or metronidazole: methotrexate, 5-fluorouracil and adriamycin.. Br J Cancer.

[OCR_00615] Twentyman P. R., Workman P. (1983). An investigation of the possibility of chemosensitization by clinically achievable concentrations of misonidazole.. Br J Cancer.

[OCR_00621] Urtasun R. C., Tanasichuk H., Fulton D., Raleigh J., Rabin H. R., Turner R., Koziol D., Agboola O. (1982). Pharmacokinetic interaction of BCNU and misonidazole in humans.. Int J Radiat Oncol Biol Phys.

[OCR_00627] WAGNER J. G. (1963). SOME POSSIBLE ERRORS IN THE PLOTTING AND INTERPRETATION OF SEMILOGARITHMIC PLOTS OF BLOOD LEVEL AND URINARY EXCRETION DATA.. J Pharm Sci.

[OCR_00643] Workman P., Twentyman P. R. (1982). Enhancement by electron-affinic agents of the therapeutic effects of cytotoxic agents against the KHT tumor: structure-activity relationships.. Int J Radiat Oncol Biol Phys.

[OCR_00650] Workman P., Twentyman P. R., Lee F. Y., Walton M. I. (1983). Drug metabolism and chemosensitization. Nitroimidazoles as inhibitors of drug metabolism.. Biochem Pharmacol.

